# Trends in adolescent secondhand smoke exposure at home over 15 years in Korea: Inequality by parental education level

**DOI:** 10.18332/tid/166132

**Published:** 2023-06-30

**Authors:** Hana Kim, Heewon Kang, Jinyoung Choi, Sung-il Cho

**Affiliations:** 1 Department of Public Health Science, Graduate School of Public Health, Seoul National University, Seoul, Republic of Korea; 2 Institute of Health and Environment, Seoul National University, Seoul, Republic of Korea

**Keywords:** adolescent, secondhand smoke, environmental tobacco smoke, parental education level, parental smoking

## Abstract

**INTRODUCTION:**

Low parental education level and parental smoking are major risk factors for household secondhand smoke (SHS) exposure among adolescents. We investigated the trend in household SHS exposure according to sex, school, and parental education level to determine whether the decline in household SHS exposure over time depends on parental education level.

**METHODS:**

We used cross-sectional Korea Youth Risk Behavior datasets (2006–2020; 806829 subjects were eligible). We applied binary logistic regression to assess household SHS exposure trends and evaluated the interaction between period and parental education level.

**RESULTS:**

Household SHS exposure over 15 years has declined. The difference (0.121) was the smallest for male middle school students with low-educated parents. The slope for the estimated probability of household SHS exposure among students with high-educated parents was steeper than that for those with low-educated parents, except for female high school students (difference=0.141). Students with low-educated parents were at higher risk of household SHS exposure (male middle school students, adjusted odds ratio, AOR=1.52; 95% CI: 1.47–1.56; male high school students, AOR=1.42; 95% CI: 1.38–1.47; female middle school students, AOR=1.62; 95% CI: 1.58–1.67; female high school students, AOR=1.62; 95% CI: 1.57–1.67). The interaction between parental education level and period was significant. We also found a significant interaction between parental education level and parental smoking (other × present interaction, AOR=0.64; 95% CI: 0.60–0.67; low–low × present interaction, AOR=0.89; 95% CI: 0.83–0.95).

**CONCLUSIONS:**

Changes in parental education level over time mainly contributed to changes in adolescents’ household SHS exposure. Adolescents with low-educated parents were at higher risk of household SHS exposure, with a slower decline. These gaps must be considered when creating and implementing interventions. Campaigns and community programs to prevent household SHS need to be emphasized among vulnerable adolescents.

## INTRODUCTION

Disparities in exposure to secondhand smoke (SHS) among adolescents should be recognized as health inequity, as they are both preventable and unfair^1^. Preventing adolescents from household SHS exposure is an imperative issue which people must take an interest in because SHS induces adverse impacts on adolescents’ health including premature death and serious diseases^2^. From 1999 to 2018, 86 of 131 countries studied reported a decline in household SHS exposure among adolescents aged 12–16 years, while 6 had a rise, and 39 reported no change^3^.

Household SHS exposure among adolescents is not low compared to exposure in public places in many countries^3,4^. The effects of smoke-free policies on household SHS exposure reduction are controversial. The number of smoke-free homes increased when public places were declared smoke-free zones by laws introduced in Australia, Canada, France, and the United Kingdom^5^. However, SHS exposure decreased significantly in cafes, restaurants, and public transportation, but not in homes after smoke-free legislation was enacted in Wales in 2007^6^.

Various risk factors for household SHS exposure among adolescents have been discovered^7^. Younger children and girls are more vulnerable to household SHS exposure^8^. Parental smoking, socioeconomic status, and education level are also major risk factors for household SHS exposure among adolescents^7,9^. The prevalence of household SHS exposure among Koreans and male smoking have declined, but both still exist (Supplementary file Figure 1).

The proportion of Koreans aged 25–64 years with tertiary education has steadily increased by 19.1% from 31.6% in 2005 to 50.7% in 2020^10^ and the average proportion of the populations of OECD countries that have received tertiary education increased by 12.9% from 2005 to 2020^10,11^. Given that a low parental education level is one of the main risk factors for child household SHS exposure, the association between decreasing household SHS exposure among Korean adolescents with higher parental education level requires confirmation.

Few studies have explored changes in adolescent household SHS exposure by temporal changes in parental education level. Thus, we explored the trend in household SHS exposure among Korean adolescents according to sex, school, and parental education level over 15 years. We also aimed to examine interaction effects between period and parental education level according to sex and school category.

## METHODS

### Data source and study participants

The Korea Youth Risk Behavior Survey (KYRBS) is a cross-sectional survey conducted by the Korea Disease Control and Prevention Agency since 2005 and enrolls approximately 60000 students (aged 12–18 years) from middle and high schools every year^12^. Data from the first survey in 2005 were excluded from our analysis because items asking about household SHS exposure among adolescents were introduced in 2006. We used pooled cross-sectional KYRBS data from 2006 to 2020 to explore household SHS exposure among never smoking adolescents. From 2006 to 2020, of 1032106 students who were enrolled, the participation rate ranged from 90.9 to 97.7% (average: 95.75%).

We used the data from never smoking Korean adolescents; a never smoker was defined as someone who answered ‘No’ to the question: ‘Have you ever smoked a cigarette, even one puff?’^13^. There were 810516 never smoking adolescents in the pooled data (1032106 adolescents). Ultimately, 806829 were eligible for this study; we excluded 3687 whose ages were not recorded.

The adolescents were divided into four groups according to sex and school: male middle school students, male high school students, female middle school students, and female high school students.

### Measures

*Outcome variable*

The outcome variable was household SHS exposure explored by asking: ‘On how many of the last 7 days have you inhaled the cigarette smoke of another at home?’. A response of ‘none’ was classified as ‘unexposed’; reports of exposure on 1–7 days were classified as ‘exposed’. The following question was used from 2006 to 2018: ‘How many days have you been at home when someone else (such as a family member or guest) smoked during the last 7 days?’. In 2019 and 2020, the question was: ‘On how many of the last 7 days have you smelled/inhaled cigarette smoke from someone else in your house?’. Caution is thus appropriate when comparing prevalence, given the changed question asked in 2019 and 2020^4^. The household SHS exposure prevalence was the number of adolescents who smelled cigarette smoke in the house over the previous 7 days (the numerator) divided by the total number of participants (the denominator).

*Independent variables*

Based on previous research, the independent variables used were sex, school, period (survey year), parental education level, lifetime alcohol consumption, perceived household economic status, academic performance, and parental smoking^7,14^. Parental education level was divided into ‘less than middle school’, ‘high school’, ‘more than college’, and ‘unknown’. We combined the educational backgrounds of both parents as: ‘High–High’, ‘Low–Low’, and ‘Other’. ‘High–High’ meant that both parents had tertiary education, and ‘Low–Low’ meant that neither had tertiary education. ‘Other’ encompassed all other possibilities, including if the father/stepfather or mother/stepmother was not included as a family member.

Lifetime alcohol consumption was classified as ‘Yes’ or ‘No’. There were 15 (continuous) period variables (2006–2020). The means of the period variables were centered to avoid multicollinearity^15^. Perceived household economic status was initially divided into ‘high’, ‘high-medium’, ‘medium’, ‘medium-low’, and ‘low’ and then simplified to ‘high’, ‘medium’, and ‘low’. Academic performance was categorized similarly. Parental smoking was classified as ‘Present’ (one or both parents smoked) or ‘Absent’ (no parents smoked; this was the classification if parents/step parents were not included as family members or did not live together).

### Statistical analysis

We used the pooled data of 15 waves to analyze the general characteristics of participants by sex and school. All analyses were weighted to reflect the survey design. No multicollinearities were detected among the independent variables used in the models. The variance inflation factor was ≤10 and the tolerance ≥0.1 in all models^16^. The household SHS exposure trends among Korean adolescents are presented over 15 years by sex, school, and parental education level.

**Figure 1:**
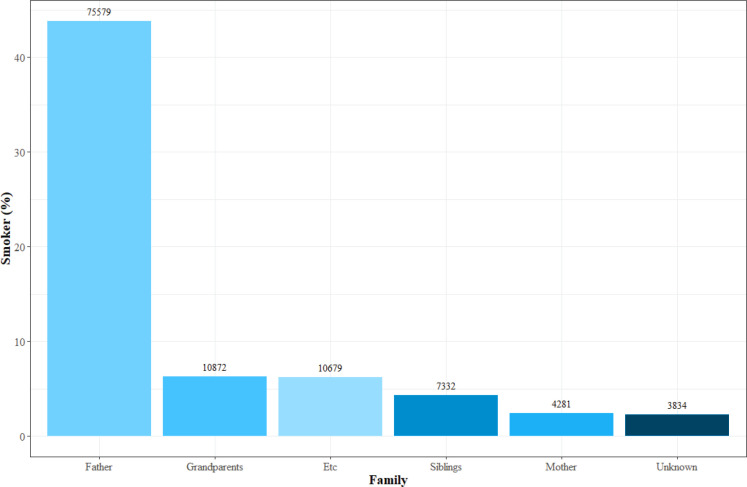
Distribution of smokers in Korean families, data from the Korea Youth Risk Behavior Web-based Survey, Cross-sectional study, 2014–2016 (N=169962)

Multiple binary logistic regression analyses were used to confirm associations between independent variables and household SHS exposure, with control of covariates. A possible interaction of period × parental education level was sought; the period was set to 5 years when confirming the dramatic change noted. We reported the results as estimated probabilities of household SHS exposure among adolescents and interaction plots for odds ratios of household SHS exposure are given for the four groups (male middle school students, male high school students, female middle school students, and female high school students) in Supplementary file Figure 2. Figure 1 shows the distribution of smokers within families. Subgroup analyses were conducted using data from 2014 to 2016 using the period variable as a continuous variable. We sought interactions between period × parental education level, period × parental smoking, and parental education level × parental smoking in the subgroup analyses.

We did not adjust for multiple comparisons following the rationale of previous researchers^17-19^. Adjusted odds ratio and 95% confidence intervals (CIs) are given. For all statistical tests, the two-tailed 5% significance level was applied (p<0.05). All statistical analyses were performed using SAS (ver. 9.4; SAS Institute, Cary, NC, USA) and figures were drawn using R (ver. 4.2.2; R Foundation for Statistical Computing, Vienna, Austria).

## RESULTS

### Descriptive results

There were 379954 male and 426875 female students. Their general characteristics are listed in Table 1. The mean age of the middle school students was 13.5 years and that of the high school students was 16.5 years. Female middle school students accounted for 26.73% of the participants, compared with 26.36% for male middle school students, 25.32% for female high school students, and 21.59% for male high school students. Of the four groups, female middle school students were most exposed to SHS (35.64%). Regarding perceived household economic status, the proportion of male middle school students who replied ‘High’ was the greatest (43.95%) and the proportion of female high school students (21.67%) who answered ‘Low’ was the highest. The proportion of both parents with college education increased as student age decreased for both male and female students. Male students self-evaluated their academic performance higher than did female students. High school students self-evaluated their academic performance lower than did middle school students. Regardless of school, the proportion of lifetime alcohol consumption was higher among males than females. Regardless of sex, high school students were more likely to have consumed alcohol than middle school students.

**Table 1:** Characteristic of the study participants, data from the Korea Youth Risk Behavior Web-based Survey, Cross-sectional study, 2006–2020 (N=806829)

*Characteristics*	*Male (N=379954)*		*Female (N=426875)*	
	*School level*		*School level*	
	*Middle (N=218337)*	*High (N=161617)*	*Middle (N=224872)*	*High (N=202003)*
**Age** (years), mean	13.51	16.47	13.53	16.48
	***n (%)^a^***	***n (%)^a^***	***n (%)^a^***	***n (%)^a^***
**Household SHS exposure**				
Yes	70274 (31.41)	46921 (28.63)	82578 (35.64)	65410 (31.59)
No	148063 (68.59)	114696 (71.37)	142294 (64.36)	136593 (68.41)
**Perceived household economic status**				
High	93221 (43.95)	49751 (31.47)	79847 (36.85)	51849 (26.65)
Medium	95583 (43.15)	77093 (47.56)	111760 (49.09)	104348 (51.68)
Low	29533 (12.90)	34773 (20.97)	33265 (14.07)	45806 (21.67)
**Parental education level**				
High-High	68283 (33.93)	47430 (30.55)	70462 (34.09)	58334 (30.50)
Low-Low	43894 (19.04)	46849 (28.06)	54840 (23.09)	67204 (31.85)
Other	106160 (47.03)	67338 (41.39)	99570 (42.82)	76465 (37.65)
**Academic performance**				
High	94937 (44.00)	62791 (38.62)	92595 (41.76)	68750 (33.64)
Medium	58415 (26.60)	47996 (29.73)	61320 (27.09)	64316 (32.05)
Low	64985 (29.40)	50830 (31.65)	70957 (31.14)	68937 (34.31)
**Lifetime alcohol consumption**				
Yes	63248 (28.99)	81153 (49.99)	58378 (25.73)	100043 (48.43)
No	155089 (71.01)	80464 (50.01)	166494 (74.27)	101960 (51.57)

a Weighted percentages.

### Trend in household SHS exposure among Korean adolescents

**Figure 2:**
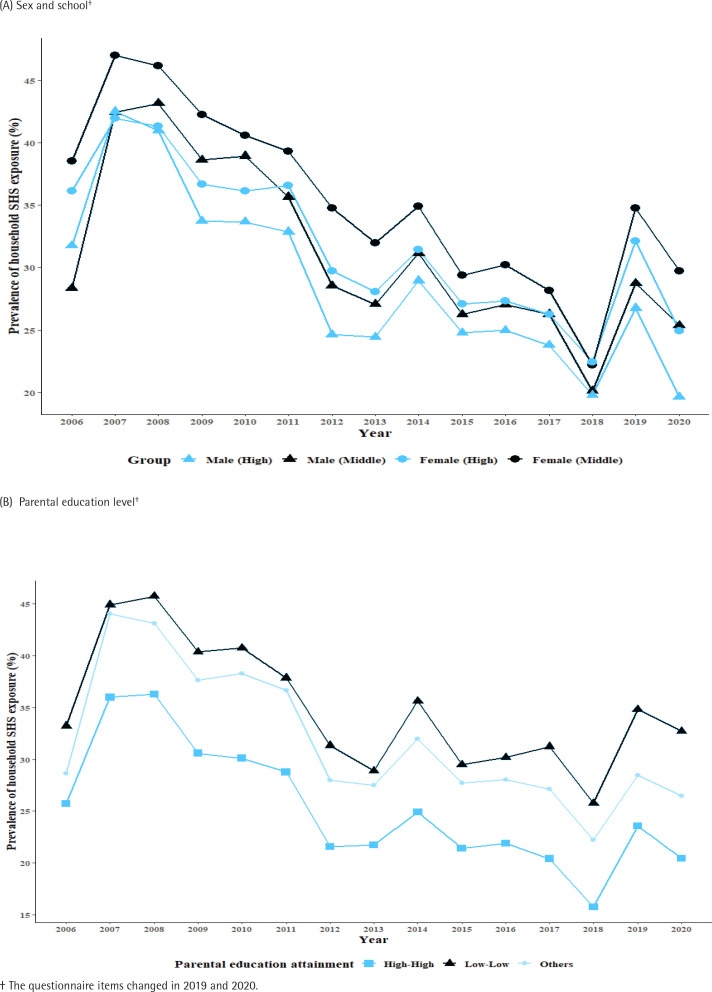
Trends in household secondhand smoke (SHS) by sex, school, and parental education level, data from the Korea Youth Risk Behavior Web-based Survey, Cross-sectional study, 2006–2020 (N=806829)

Figure 2 shows the trend in household SHS exposure stratified by sex, school, and parental education level. The trend in household SHS exposure over 15 years varied by sex and school. Female middle school students had the highest exposure at home over the 15 years. The prevalence of household SHS exposure among Korean adolescents has been declining, but remains higher when parents are poorly educated compared to well-educated.

### Results of multiple binary logistic regression with an interaction term

The covariate-adjusted multiple binary logistic regression models with an interaction term are shown in Table 2. Models with and without interaction terms (period × parental education level) are presented by the groups. In a model without an interaction term, the period coefficient suggested a 23% decreased odds of household SHS exposure in the 5 years after 2006 (AOR=0.77; 95% CI: 0.76–0.78) among male high school students (Model 3). However, the period coefficient revealed a 17% decreased odds of household SHS exposure in the 5 years after 2006 (AOR=0.83; 95% CI: 0.82–0.85) among female high school students in the model with the interaction term (Model 7). The simple effect of low parental education level remained significant, with about a 62% increased odds of household SHS exposure among female students whose parents both lacked tertiary education (regardless of school) compared to female students with parents who both had received tertiary education (female middle school students: AOR=1.62; 95% CI: 1.58–1.67; female high school students: AOR=1.62; 95% CI: 1.57–1.67) (Models 5 and 7).

**Table 2:** Coefficients derived using multiple logistic regression models of household secondhand smoke exposure by sex and school level among non-smoking adolescents, data from the Korea Youth Risk Behavior Web-based Survey, Cross-sectional study, 2006–2020 (N=806829)

*Variable*	*Male (Middle)^c^ (N=218337)*		*Male (High)^c^ (N=161617)*		*Female (Middle)^c^ (N=224872)*		*Female (High)^c^ (N=202003)*	
*Model^a^*	*1*	*2*	*3*	*4*	*5*	*6*	*7*	*8*
	*AOR (95% CI)*	*AOR (95% CI)*	*AOR (95% CI)*	*AOR (95% CI)*	*AOR (95% CI)*	*AOR (95% CI)*	*AOR (95% CI)*	*AOR (95 % CI )*
**Period^b^**	0.81 (0.80–0.82)***	0.77 (0.76–0.79)***	0.77 (0.76–0.78)***	0.74 (0.72–0.77)***	0.82 (0.80–0.83)***	0.77 (0.75–0.79)***	0.83 (0.82–0.85)***	0.80 (0.77–0.82)***
**Parental education level** (Ref. High-High)^d^								
Other	1.29 (1.26–1.32)***	1.28 (1.25–1.31)***	1.28 (1.24–1.32)***	1.27 (1.23–1.31)***	1.37 (1.33–1.40)***	1.36 (1.33–1.40)***	1.41 (1.37–1.45)***	1.40 (1.36–1.44)***
Low-Low	1.52 (1.47–1.56)***	1.53 (1.49–1.58)***	1.42 (1.38–1.47)***	1.43 (1.38–1.48)***	1.62 (1.58–1.67)***	1.62 (1.57–1.66)***	1.62 (1.57–1.67)***	1.61 (1.57–1.66)***
**Interaction term**								
Period × Other		1.06 (1.03–1.09)***		1.04 (1.00–1.08)*		1.09 (1.05–1.12)***		1.07 (1.03–1.11)***
Period × Low-Low		1.09 (1.05–1.13)***		1.07 (1.03–1.12)**		1.06 (1.03–1.10)**		1.06 (1.02–1.10)**

a AOR: adjusted odds ratio; adjusted for age, perceived household economic status, lifetime alcohol consumption, and academic performance.

b Over the 5 years.

c Middle: Middle school student. High: High school student.

d Parental education level: High-High, both parents with at least a college degree; Low-Low, both parents with no college degrees; Other, no information on parents’ educational level or parents’ educational levels are different from each other.

*** p<0.001,

** p<0.01,

* p<0.05.

However, low parental education level increased the odds of household SHS exposure among male high school students by about 42% over the 5 years (AOR=1.42; 95% CI: 1.38–1.47) (Model 3). For male middle school students with low-educated parents, compared to those whose parents had received tertiary education, there was an odds difference of about 1% coefficient between models with and without the interaction term (Models 1 and 2), but there was no difference between models with and without the interaction term for female middle school students (Models 5 and 6). All interactions between parental education level (Low-Low and Other) and period over the 5 years were significant.

**Figure 3:**
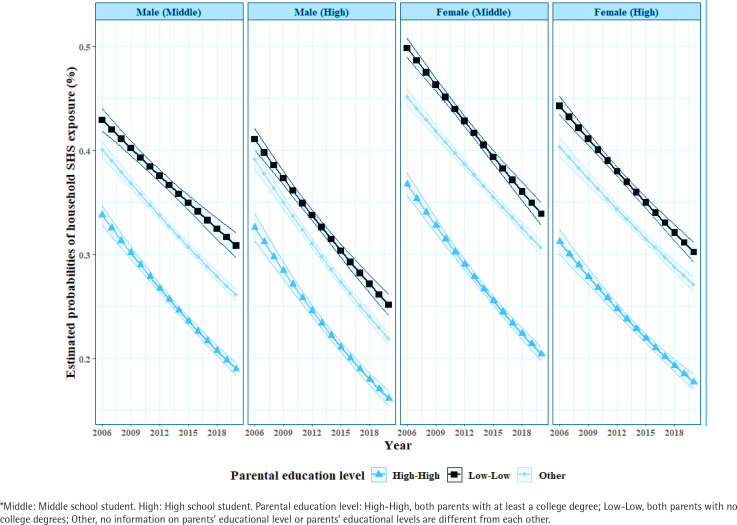
Interaction plot for estimated probabilities of household secondhand smoke exposure between period and parental education level by the groups*, data from the Korea Youth Risk Behavior Web-based Survey, Cross-sectional study, 2006–2020 (N=806829)

The interaction between period and parental education level is described by estimated probabilities of household SHS exposure among adolescents. The interaction plots of trends in household SHS exposure over 15 years by the groups defined by sex and school are shown in Figure 3. Table 3 gives the difference in estimated probabilities of household SHS exposure by sex, school, and parental education level between 2006 and 2020. Except for female high school students, Korean students with highly educated parents experienced a greater decline in household SHS exposure over the 15 years than students with low-educated parents. The difference in estimated probability of household SHS exposure between 2006 and 2020 among female high school students for whom both parents had at least a college degree was 0.135, while the difference was 0.141 for female high school students for whom both parents did not have college degrees. The group with the smallest decline between 2006 and 2020 was male middle school students with low-educated parents (difference = 0.121).

**Table 3:** Difference in estimated possibilities of household secondhand smoke exposure by sex, school and parental education level among adolescents, data from the Korea Youth Risk Behavior Web-based Survey, Cross-sectional study, 2006–2020 (N=806829)

*Group^a^*	*Parental education level^b^*		
	*High-High*	*Low-Low*	*Other*
Male (Middle) (N=218337)	0.148	0.121	0.140
Male (High) (N=224872)	0.165	0.159	0.173
Female (Middle) (N=161617)	0.163	0.160	0.145
Female (High) (N=202003)	0.135	0.141	0.132

a Middle: Middle school student. High: High school student.

b Parental education level: High-High, both parents with at least a college degree; Low-Low, both parents with no college degrees; Other, no information on parents’ educational level or parents’ educational levels are different from each other.

### Subgroup analyses

Interactions between parental smoking, parental education level, and the period were confirmed (Table 4). The period × parental education level interaction was not significant regardless if parental smoking was controlled for (period × other interaction odds ratio, AOR=1; 95% CI: 0.97–1.03; period × low–low interaction AOR=0.99; 95% CI: 0.95–1.03) (Model 2). Likewise, the parental smoking × period interaction was not significant after adjusting for parental education level (period × present interaction AOR=1.03; 95% CI: 1.00–1.06) (Model 3). In other words, parental smoking did not affect household SHS exposure among students over time. However, the parental smoking × parental education level interaction was significant after controlling for period (present × other interaction AOR=0.64; 95% CI: 0.60–0.67; present × low –low interaction AOR =0.89; 95% CI: 0.83–0.95) (Model 4).

**Table 4:** Estimates from descriptive analyses and multiple logistic regression models of household secondhand smoke exposure among non-smoking adolescents, data from the Korea Youth Risk Behavior Web-based Survey, Cross-sectional study, 2014–2016 (N=169962)

*Variable*	*Model 1^a^*	*Model 2^a^*	*Model 3^a^*	*Model 4^a^*
	*AOR (95% CI)*	*AOR (95% CI)*	*AOR (95% CI)*	*AOR (95% CI)*
**Period**	0.92 (0.90–0.94)***	0.93 (0.90–0.95)***	0.91 (0.89–0.93)***	0.92 (0.91–0.94)***
**Parental education level** (Ref. High-High, N=60869)^b^				
Other (N=71281)^b^	1.36 (1.33–1.40)***	1.39 (1.35–1.43)***	1.39 (1.35–1.43)***	1.78 (1.71–1.85)***
Low-Low (N=37812)^b^	1.60 (1.55–1.65)***	1.37 (1.32–1.41)***	1.37 (1.32–1.41)***	1.46 (1.38–1.54)***
**Parental smoking** (Ref. Absent, N=105310)^c^				
Present (N=64652)^c^		6.42 (6.26–6.58)***	6.43 (6.27–6.59)***	8.06 (7.72–8.40)***
**Interaction term**				
Period × Other	1 (0.97–1.03)	1 (0.97–1.03)		
Period × Low-Low	0.98 (0.94–1.01)	0.99 (0.95–1.03)		
Period × Present			1.03 (1.00–1.06)	
Other × Present				0.64 (0.60–0.67)***
Low-Low × Present				0.89 (0.83–0.95)**

AOR: adjusted odds ratio.

a All models were adjusted for age, sex, school level, perceived household economic status, lifetime alcohol consumption, academic performance, and additionally adjusted for: Model 2, parental smoking; Model 3, parental education level; and Model 4, period.

b Parental education level: High-High, both parents with at least a college degree; Low-Low, both parents with no college degrees; Other, no information on parents’ educational level or parents’ educational levels are different from each other.

c Parental smoking, Absent: no parent smoking; Present: one or both parents smoking.

*** p<0.001,

** p<0.01,

* p<0.05.

## DISCUSSION

The household SHS exposure of Korean adolescents decreased over 15 years and varied by sex, school, and parental education level. The trends in estimated probabilities of household SHS exposure differed by parental education level. Male middle school students with low-educated parents showed the smallest change in estimated probability of household SHS exposure among adolescents over 15 years among the four groups. We found that temporal changes over the 15 years in parental education background affected adolescent household SHS exposure: adolescent household SHS exposure decreased when parental education level increased. The interaction between parental smoking and parental education level was significant, while the interaction between period and parental smoking was not significant over a 3-year interval.

Our findings showed different trend slopes of the probabilities by parental education level. Except for female high school students, students with high-educated parents had a significantly larger decline in household SHS exposure than students with low-educated parents. We interpreted this as being caused by changes in education inequality over time. Parental education level could be an essential factor in children’s health^20^. An annual increase in parental education level was associated with a lower risk of mortality among children^20^. The weaker association of parental education with children’s health in developing countries over time could be explained by changes in the quality of parental education or living standards as confounders, and by changes in income or social environment as mediators^20^.

Differences in household SHS exposure among adolescents according to period and parental education level have been reported^21,22^. Gagne et al.^21^ found a substantial decline in household SHS exposure among Canadian non-smoking adolescents, but no significant interaction between period and household education level. Pisinger et al.^22^ found that the decline in parental smoking at home did not differ significantly according to parental education level in Denmark. The difference between our findings and previous studies may be explained by the rapid increase in tertiary education in Korea (19.1%; from 31.6% in 2005 to 50.7% in 2020) compared to Canada (14%, 46–60%) and Denmark (5.8%, 33.5–39.3%)^10^. The length of data may also be a reason for the significant interaction between period and parental education level. A previous study used data from 2013 to 2018^21^, while we used data from 2006 to 2020, to explore changes in household SHS exposure^21^.

We found that the risk of Korean household SHS exposure among female middle school students with poorly educated parents decreased the most from 2006 to 2020. Young female students with low-educated parents were the group most likely to be exposed^7,8,23^. Non-smoking Korean female students were more likely to be exposed to SHS at home than males because female students spent more time with their smoking parents^8^. Younger adolescents were more likely to be exposed than older adolescents because high school students finish school later than middle school students^8^. A study of NHANES data from 2003 to 2014 suggested that, as children get older, they spend less time at home with their parents and more time in parks or school with their friends, regardless of whether their parents smoke^23^. The decrease in SHS exposure over the last 15 years in the most susceptible group could be attributed to the implementation of tobacco control policies in Korea^24^. After the Health Promotion Act was passed in 1995, the Korean government designated public places as smoke-free areas and ran campaigns to promote smoking cessation as a social norm to prevent SHS exposure^24^.

However, differences in household SHS exposure by parental education level indicate blind spots in tobacco control policies that do not consider vulnerable groups^21^. Prioritizing the prevention of household SHS exposure in vulnerable groups is essential because such inequalities may lead to health problems over the life course^1^. Existing policies and media campaigns may be less effective in low-educated parents. We found that household SHS exposure decreased the least among male middle school students with low-educated parents. Current population-level interventions have limitations. Therefore, policies and interventions for preventing household SHS exposure should target vulnerable groups.

Smoke-free areas do not directly reduce household SHS exposure^25^, but do lower smoker numbers, encourage implementation of smoke-free home rules, and create new social norms^26^. A review of studies in the UK, the US, Australia, and New Zealand showed that comprehensive tobacco control programs reduced smoking at home, but direct effects in terms of smoke-free homes were less apparent^26^. However, media campaigns change smoking behaviors at home and the social norms of SHS^26^. Strong smoke-free laws for workplaces and restaurants increased voluntary smoke-free home rules, applied by both people who smoke and those who do not^27^.

Parental education level is positively associated with imposition of smoke-free home rules, in turn reducing child SHS exposure. Of parents with unmarried children, 84.9% of those educated to lower than high school level, 86.7% of those who graduated from high school, and 96.8% of those with college or higher education, set smoke-free home rules^28^. One or both smoking parents with children aged <18 years were more likely to enforce smoke-free home rules if they graduated from high school compared to less than high school education^29^. In addition, parents with college or higher education in the US were more likely to impose smoke-free home rules than were less educated parents^29^.

In our subgroup analysis, the interaction between parental smoking and parental education level was significant, indicating that the rates of parental smoking for students with high-educated parents differed from that of those with low-educated parents. Zheng et al.^25^ found a reduction in paternal smoking prevalence as paternal education level increased. The best way to reduce adolescents’ household SHS exposure is to improve parental education, but this is difficult and time-consuming^25^. Therefore, smoking parents must have access to smoking cessation services, as well as regular health education^25^. Both parents and students should receive SHS prevention education related to application of the completely smoke-free rule at home. Education benefits the health of students whose parents are less educated^30^. SHS prevention education for adolescents may offset the negative health effects of having low-educated smoking parents^30^.

To reduce household SHS exposure, not only smoke-free home rules but also campaigns and community programs for parents are urgently needed to create social norms for smoke-free environments in society as a whole. Interviews with smoking Israeli parents with children aged <7 years revealed that the parents were aware of the health risks associated with SHS, but were confused about preventative methods and whether smoke-free home rules were effective^31^. Therefore, it is necessary to explain the need for smoke-free homes or to deliver the message via a campaign^31^. A randomized controlled trial on the effectiveness of household SHS interventions by community health workers found that smoking parents or caregivers with children, adopted complete smoking bans in China after the smoking hygiene intervention^32^. Hence, community programs that seek to establish smoke-free households raise and sustain awareness of the risks of SHS exposure among family members^33^, and are especially effective if the parents are not well-educated.

### Strengths and limitations

This study was unique in that it investigated the trend in household SHS exposure among Korean adolescents over 15 years and its interaction with parental education level. However, it had several limitations. First, it was not possible to analyze the parental smoking variable over the entire 15-year period, which is one of the most important variables related to adolescents’ exposure to SHS at home, due to the limitations of the secondary data. Second, the KYRBS data used in this study may have been influenced by both recall and social desirability bias^34^. The prevalence of household SHS exposure may have been underestimated compared to a biomarker, since it relied on self-report measures^35^. Also, the adolescents may not have accurately reported their parental education level. However, our findings are useful because they facilitate targeted public health interventions for groups at particular risk of household SHS exposure, as indicated by the different declining trends by parental education level.

## CONCLUSIONS

Household SHS exposure among Korean adolescents was affected by adolescents’ sex and school, and parental education level. We found an interaction between period and parental education level. The decline in SHS exposure was significantly slower among adolescents for whom the education level of both parents was low. Careful programs and policies may prevent adolescent household SHS exposure. Smoke-free home rules and prevention education, and campaigns targeting vulnerable groups, may further reduce household SHS exposure in the future.

## Data Availability

The data supporting this research are available from the authors on reasonable request.
