# Supplementary material for: Trends in adolescent secondhand smoke exposure at home over 15 years in Korea: Inequality by parental education level
Source: Tob Induc Dis. 2023 Jun 30;21:88. doi: 10.18332/tid/166132 (PMC10311469; doi:10.18332/tid/166132)
Supplement: Supplementary file 1 [file TID-21-88-s1.pdf]

Supplement Figure 1. Trends in the smoking and exposure to secondhand smoke of Koreans from 2006 to 2020. Data from Korea Youth Risk Behavior Web-based Survey and Korea National Health and Nutrition Examination Survey.

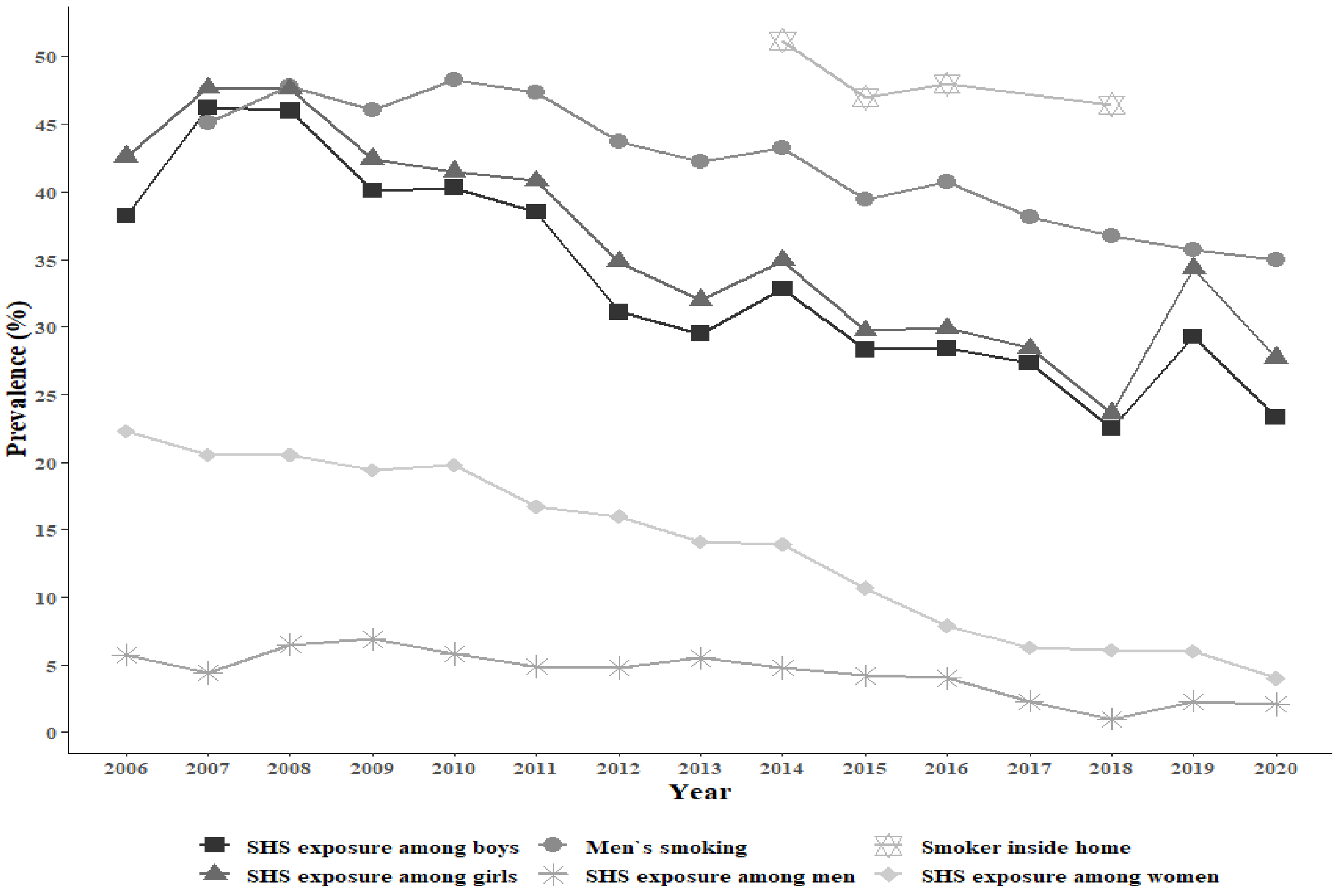

References

1. Ministry of Education, Ministry of Health and Welfare, Korea Centers for Disease Control and Prevention. The statistics on the 17th Korea youth risk behavior survey in 2021. In Korean. Korea Centers for Disease Control and Prevention; 2022.

2. Korea Centers for Disease Control and Prevention. Health statistics 2021: Korea National Health and Nutrition Examination Survey; 2022. Accessed March 25, 2023. http://www.cdc.go.kr

Supplement Figure 2. Interaction plot for odds ratios of household secondhand smoke exposure from 2006 to 2020 among Korean adolescents, Data from Korea Youth Risk Behavior Web-based Survey, Cross-sectional study (N=806829)

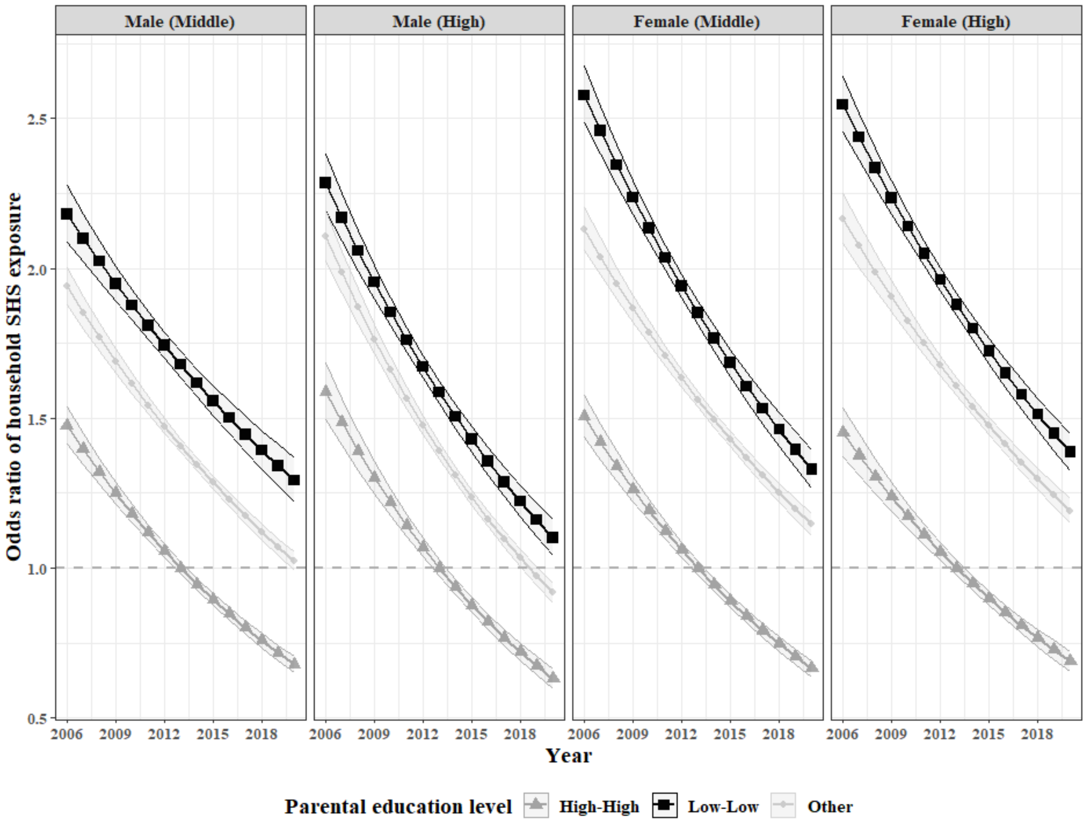

[*] Middle: Middle school student, High: High school student, Parental education attainment: (High-High) Both parents with at least college degrees, (Low-Low) Both parents with no college degrees. (Other): No information on parents' educational background or parents' educational background is different from each other.

© 2023 Kim H. et al.
